# Experiences of Online Sexual Violence: Interviews With Swedish Teenage Girls in Psychiatric Care

**DOI:** 10.1177/10778012231203000

**Published:** 2023-09-21

**Authors:** Frida Carlberg Rindestig, Katja Gillander Gådin, Inga Dennhag

**Affiliations:** 1 Child- and Adolescent Psychiatry, Department of Clinical Science, Umeå University, Umeå, Sweden; 2 621629 Department of Health Sciences, Mid Sweden University, Sundsvall, Sweden

**Keywords:** youth, child- and adolescent psychiatry, online victimization, thematic analysis, feminism, poly victimization

## Abstract

Research about online sexual violence (OSV) is needed to be able to better meet the needs of girls in psychiatric care. The objectives of this study are to explore experiences of online sexual violence among young female psychiatric service users. Interviews with nine girls with psychiatric care needs were analyzed with thematic analysis. The findings are summarized in four themes which contribute to the notion that online sexual violence is only one, albeit important, part of a more complex picture of violence among young girls in psychiatric care. The girls’ narratives are shaped by, as well as reproducing gender norms.

The technological development of the internet and smart devices has in many ways been positive, but has also created more arenas where sexual violence can take place. In recent decades, research of sexual violence has expanded and now also consider online arenas (Jonsson et al., 2019; Svedin & Jonsson, 2017; Zetterstrom Dahlqvist & Gillander Gadin, 2018). The modi operandi for online sexual violence include, but are not limited to, repeated requests for nude pictures, dickpics, online publication of nude pictures, and unwanted sexual advances such as unwanted solicitation, online grooming, and online rape.

Sexual violence is defined by the World Health Organization (WHO) as “any sexual act, attempt to obtain a sexual act, unwanted sexual comments or advances, or acts to traffic, or otherwise directed, against a person's sexuality using coercion, by any person regardless of their relationship to the victim, in any setting, including but not limited to home and work.” (WHO, 2012). It is regarded as a serious global public health problem, and can be perpetrated in different arenas, and in different modalities. In this article, we have used a broad definition of online sexual violence that includes a range of practices and events that have the common aspects of; being unwanted or perceived in a negative way by the subjected person; of a sexual nature; and perpetrated through some kind of digital media.

The prevalence of online sexual violence depends on how it is conceptualized and the method of measurements. Online sexual harassment, which is defined as being subjected, via electronic means to, for example unwelcome sexual comments, jokes, or gestures, or being asked to do something sexual when one does not want to, is thought to affect around 15% of American teenagers (Copp et al., 2021). A specific kind of online sexual harassment that has become ubiquitous in the present time is receiving unrequested pictures of male genitals, the so-called dickpic (Ringrose et al., 2021). The prevalence of receiving a dickpic among youth is not extensively researched, but some research suggests that for women who had at one time received a consensual dickpic, 90% had also received unwanted dickpics (Marcotte et al., 2021).

Another form of online sexual harassment is unwanted requests or pressure to send nudes, so-called pressurized sexting. This is often tightly interwoven with the practice of consensual sexting (Ringrose et al., 2013, 2022; Thomas, 2018; Thorburn et al., 2021). The prevalence of sexting was around 20% in a Swedish sample of youth from 2014 but this had increased to 37% by 2021 (Jonsson et al., 2014; Svedin et al., 2021). A meta-analysis of four studies from Canada, the US, Europe, and the Czech Republic found a prevalence of having one's sext furthered without consent was 8.4% (Madigan et al., 2018), something that some researchers propose should go under the label of image-based sexual abuse (McGlynn et al., 2017)

There is also the case of more severe forms of online sexual violence, which is often referred to as online sexual abuse, or when the survivor is underaged: online child sexual abuse. The prevalence of online child sexual abuse among youth in a large community school sample in Sweden was around 0,5% (Svedin & Jonsson, 2017).

Although it can affect both men and women, boys and girls, the global prevalence of sexual violence is around three times higher for girls and women than it is for boys and men (Borumandnia et al., 2020). The same goes for sexual violence in online arenas (Jonsson et al., 2019; Ståhl & Dennhag, 2021; Zetterstrom Dahlqvist & Gillander Gadin, 2018).

## Online Sexual Violence Through a Gender Lens

In our view, a gender perspective is used to reject individualizing the problem of sexual violence, and instead acknowledge the structural underpinnings of the phenomenon. Sexual violence, in whichever arena it is perpetrated, can be understood as happening on a continuum, from enticement, to coercion, to force. This suggests that all acts of sexual violence, online or offline, are to be seen as serious, and a part of a structural societal problem of gender inequality (Kelly, 1987). The reason that sexual violence is perpetrated is hence to produce and reproduce certain masculinities and femininities, upholding certain gender hierarchies (Connell, 2012; Tolman et al., 2016). Societal norms of heterosexuality, such as the sexual double standard, posits that males should be sexually active, assertive, and sexually dominant, while females should be sexually passive and submissive and have less desire. This normalizes male sexual aggression and de-normalizes sexual activity in females (Sanchez et al., 2012).

The relevance of a gender perspective for psychiatric practice, especially regarding girls in need of psychiatric care, is that it acknowledges social inequalities as an important factor in mental health difficulties. Gender is of particular significance for mental health because it permeates our lives through to the very core of our personality and shapes our life circumstances. Gender also shapes how behaviors and emotional states are viewed and what is considered healthy for girls or boys, which has implications for the assessment of mental health among women and girls in general, and girls in psychiatric care specifically (Tew, 2005). In the case of psychiatry, gender can thus be seen as intersecting with mental health difficulties through several pathways, such as gender patterns of the prevalence of sexual violence, but also through norms and discourses shaping both the experiences of sexual violence victims and the societal responses to their distress. In researching online sexual violence among girl psychiatric patients, care should thus be taken to not essentialize the findings as part of core mental health difficulties, reinforcing a stigmatized role of girls who are sexually victimized where victims can be blamed and pathologized (Connell, 2012; Kelly, 1987; Tolman et al., 2016).

## Girls in Psychiatric Care

A significant body of research suggests that being exposed to different forms of sexual violence is closely correlated with psychiatric morbidity and psychiatric service use (Chen et al., 2010; Hauw et al., 2020). In the child and adolescent psychiatric population, the prevalence of interpersonal violence, including sexual violence, is estimated to be higher than in the general population (Hultmann & Broberg, 2016; Oram, 2020). One study of a psychiatric sample shows that also online sexual violence is highly prevalent in this group (Dönmez & Soylu, 2019). Regarding sexual violence specifically, research shows that being exposed to sexual abuse increases suicidality and the need for psychiatric care services among young girls (Rajan et al., 2020). A growing body of research connects online sexual violence to different kinds of mental health problems in youth and is therefore a risk factor for psychiatric service needs (de Santisteban & Gamez-Guadix, 2018; Joleby et al., 2020; Jonsson et al., 2019; Svedin & Jonsson, 2017; Whittle et al., 2013; Zetterstrom Dahlqvist & Gillander Gadin, 2018). Research suggests that the consequences of online sexual violence do not differ much from the consequences seen in survivors of other forms of sexual violence. However, there seem to be specific characteristics of online sexual violence, that differentiate it from violence perpetrated IRL, such as the permanence and resurfacing of documented abuse online which can retraumatize victims (Hamilton-Giachritsis et al., 2020).

Risk factors for being exposed to sexual violence—whether it is online or IRL—are being young, being a woman, having some kind of disability, having experiences of sexual abuse in childhood, and living in poverty (Oram et al., 2017). Several of these factors are also risk factors for mental disorders, thus highlighting the social determinants of both exposure to sexual violence and mental health difficulties.

There is a lack of research about girls in psychiatric care in general, and specifically regarding the connections between sexual violence, sexuality, and mental health. However, studies have been conducted in populations that overlap with girls in psychiatric care, namely girls in societal care such as detention facilities and secure homes. In these contexts, girls’ sexuality is shown to be neglected, and the girls are constructed by the staff as being without sexual agency. That is, either they are viewed as a-sexual, or only as victims of sexual abuse (Henriksen, 2017; Overlien, 2003).

## Qualitative Studies of Online Sexual Violence

Qualitative studies of online sexual violence among youth have been conducted from two epistemologically different perspectives; one where psychological consequences are emphasized (Hamilton-Giachritsis et al., 2020; Joleby et al., 2020; Leonard, 2010) and another, constructionist perspective where societal discourses and norms are highlighted (Ringrose et al., 2022). However, to our knowledge, no qualitative study has been done on the phenomenon of online sexual violence in a population of girls in need of psychiatric services from a constructionist, gender perspective.

## Knowledge Gap

In summary, the above-mentioned research indicates that online arenas of sexual violence should be considered when assessing the total burden of violence among girls in psychiatric care. This is because they are known to be at risk for interpersonal violence, more often poly-victimized, and in general more vulnerable due to the psychological distress they already experience. There is also a need to approach this subject from a gender point of view. In psychiatric research there is often an overemphasis on essentialist perspectives (Adriaens & De Block, 2013). With a gender perspective we want to counteract the essentialization of victimhood that young girls in psychiatry who are subjected to online sexual violence can otherwise suffer (Phillips, 2010).

## Aims of the Study

This study aims to explore the experiences of online sexual violence among young female psychiatric service users from a gender perspective. From a gender perspective all acts of sexual violence, online or offline, are to be seen as serious, and part of a structural societal problem of gender inequality (Kelly, 1987). Listening to young girls’ voices about this topic could give insights into how interventions from child and adolescent psychiatric services regarding the topic of online sexual violence could be tailored to their needs.

## Method

Ethical approval was sought and approved by the Ethical Review Authority (reference number 2020-02309). We used reflexive thematic analysis as a method to guide data collection and analysis (Braun & Clarke, 2021).

Eligible for the study were young people with a current or earlier contact with psychiatric services in the age range of 15–20 years of age who acknowledged experience of some kind of online sexual violence, from online sexual harassment to online sexual abuse or assault. No diagnostic labels were used as exclusion or inclusion criteria. However, young people with for instance active psychosis or severe suicidality were deemed too vulnerable to participate.

We aimed at cooperating with therapists at a child and adolescent psychiatric clinic in northern Sweden who could ask the young people in treatment to join the study. In the end, however, most participants were recruited from an earlier quantitative study at the clinic. Participants who in the earlier study acknowledged experience of unwanted online sexual harassment were contacted. In some instances, snowball sampling was also used. The participants were initially contacted via SMS or email, and if they agreed to participate, we decided on a time and place for a first meeting. The first meeting served as a preparation for the interview, with room for questions, filling in a short questionnaire, and building rapport. Informed consent was obtained both orally and in written form when the girls filled in the first questionnaire. The questionnaire consisted of questions about history of sexual violence and the Juvenile Victimization Questionnaire which measures a wide range of interpersonal victimization events (Finkelhor et al., 2005a, 2005b). This had the purpose of collecting some background information about the participants without making the interview too long.

Due to the COVID-19 pandemic, and to be able to recruit participants from outside the city, the participants could choose either to participate via video link or to meet face-to-face.

The interviews were conducted by the first author, who is a licensed psychologist with experience of trauma therapy in a child and adolescent psychiatric setting with victims of sexual violence. The interviews were conducted in a non-formal manner with a lot of follow-up questions and prompts. The interviewer was monitoring the participant's psychological status throughout, and no participant expressed heightened distress during or after the interview. The informants got information that in need they could contact the interviewer after the interviews. Most of the participants expressed that the interview had been a good experience, and none needed any contact afterwards. The interviews were audio-recorded, and the sound files were transferred to a safe online storage. Verbatim transcriptions of the interviews were made by an external company. Confidentiality was ensured by using codes for the material and recordings.

### Participants

Nine participants were recruited. They were between 15 and 19 years of age and had a range of experiences of online sexual violence (mean age  =  17.33, SD  =  1.33). Some of the girls had experiences of severe and traumatic online as well as offline sexual abuse, while other girls had experiences that were limited to involuntary sexual exposure and repeated requests for nudes from acquaintances and strangers. All of the participants had multiple victimization experiences in their biography (mean number of events was  =  15, SD  =  5,18). With the definition of poly-victimization being >7 events, all the participants were considered poly-victimized (Finkelhor et al., 2005a, 2005b). None of the participants did seek psychiatric care primarily due to online sexual violence, and they had a range of different psychiatric issues such as anorexia, depression, and anxiety. Co-morbidity was high. The reason for this study was limited to nine participants were mainly due to recruitment difficulties. We also perceived a saturating in the themes included when we had analyzed about two thirds of the transcripts, which supported the decision to not make further efforts in recruiting.

### Data Analysis

Our point of departure was reflexive thematic analysis according to Braun & Clarke (Braun & Clarke, 2006, 2022). After the first read-through of a transcript, we did a preliminary coding. Thereafter we read and coded the next interview. We approached the material with a latent focus, which means that implicit ideas and or concepts that underpin the explicit accounts were analyzed. During the coding process, we constructed broader themes which were then revised and refined several times during the writing process (Braun & Clarke, 2006, 2022). In this article, our epistemological point of departure was constructionist, viewing knowledge as situated and partial (Gergen, 2015). The girls’ narratives were hence not thought of as objectively reflecting the world or their identities and social relations, but seen as constructions depending on both the micro context with the interviewer, and larger societal discourses of the topics discussed.

To strengthen the analysis, we had discussions about the codes and themes in our research group, and when themes were generated, we consistently went back to the transcripts of the interviews to see that we did the material justice. Generating the themes was thus an iterative process. We critically reflected on our roles as researchers, and our research practice and process as well as our preunderstanding of the topic. Two of the authors (FCR and ID) are psychologists working in child and adolescent psychiatry with trauma therapy, the third author (KGG) is a researcher with young people and victimization as a special focus.

## Findings

### Entangled in a Web—The Continuums of Online Sexual Violence

In the girls’ narratives, we saw three kinds of continuums of experience, expressed as subthemes, regarding online sexual violence: From nagging to online rape, Online/Offline—same but different, and Violence in the plural. These three continuums can be thought of as a web—in which the girls were entangled. See Table 1.

**Table 1.** Findings From the Thematic Analysis of Interviews with Teenage Girls in Psychiatric Care About Experiences of Online Sexual Violence.

Themes	Entangled in a web—the continuums of online sexual violence	Making sense of online sexual violence	Being affected by online sexual violence	Resisting online sexual violence
Subthemes	From nagging to online rape Online–offline, same but different Violence in the plural	Fulfilling unmet needs Feeling pressured and coerced Acknowledging curiosity	Psychiatric symptoms Coloring the view of men A commonplace experience	Shutting the door Fighting back

#### From Nagging to Online Rape

This subtheme describes the sliding scale between different forms of sexual violence. The range of online sexual violence events that the girls described was broad and included many different types of acts and interactions in a range from being nagged on, sending sexual images and receiving sexually explicit pictures, to online-facilitated sexual abuse. All the girls had experiences of being sent unrequested pictures of male genitals, so-called dickpics. These could come from both strangers and acquaintances and were always framed as unwanted and disturbing.

Several of the girls describe that the sexual interactions they were involved in online, primarily via social media, often began with requests that were thought of as “innocent” and consensual at first but escalated to more and more uncomfortable ones.

A few of the girls described experiences of extortion, with threats to publish explicit images or films. Several of the girls also had experiences of being raped, either in real life (IRL) or online, and some had also been offered and accepted different kinds of payment in exchange for sex, such as drugs or money. One girl described being pressed, lured, or coerced online into meetups for IRL sexual encounters:

Erin (19): So I think, they just wrote, do you want to meet up? And I was like, no I can’t manage right now. But they were like, please. ‘Cause I know you like it! And I was like, yeah I do. At least I thought that at the time. (…) And it was like; do you wanna have sex? Don’t you want to have sex for drugs? When you were in it, it was like, why not. I need, I have to have something.

What many of these online experiences had in common was the risk of photos or films of the girls’ naked bodies ending up in the wrong hands. On the question about what would happen if the sent photos came out in public, one of the girls answered:

Agnes (17): Well. Today's schools or school pupils are quite judging I would say. So you would probably be judged as a whore or something like that. (…) And then you might lose some friends, or I mean no one wants to be friends with a whore.

#### Online–Offline—Same but Different

This subtheme describes how the digital and IRL arenas are both seen as intertwined and having separate qualities. In the girls’ narratives, it was clear that their experiences of sexual violence were moving back and forth between the online and offline spaces, and more often than not, the girls had been exposed in both modalities. Online life was an inseparable part of the girls’ social life, which meant that they had to take into account the pros and cons of online space. To be able to socialize in the way they want they had to use platforms and social media in which they were constantly required to handle sexual requests or images. Paradoxically, the girls described the online world as having unique features compared to IRL. In the girls’ view, the online space seems to make possible some behavior that the offline world does not. The girls expressed that what they saw as the main difference between the online and the offline space was the anonymity that the online space offers and that this seemed to facilitate the abundance of unwanted sexual activity there. Some of the girls felt that they were more exposed in the online modality than IRL and described the online space as a lawless land, in which no one could see what was happening:

Anna (17): Ehm, online you need proof. I mean more proof. IRL if someone raped you or did something like that, you could like, I mean you could get a bruise or something. Or someone could see. (…) But online no one may be able to do anything about it. I mean you have to have proof for the cops to be able to do anything about it.

In contrast, some girls described the physical features of the offline modality as making sexual violence tangible, and thus more real, than in the online modality. As Siri, 17 years, expressed: “But, also. I think it's a hell of a lot worse to like being raped or being subjected to a flasher or something in real life than it is to get a dickpic online.”

Another feature that seemed to distinguish the online space was the vastness of the web and the permanence of visual material once sent via digital media. The girls who had sent sexual images could not be sure where the pictures were and who had access to them. This felt threatening and overwhelming. One of the girls described the lack of control she felt concerning the pictures:

Anna (17): Yeah, and then you get like, ehm paranoid sort of. Like has he put this on a porn site? Is that why that old man is looking at me a bit strange? You know, it gets like…(…) That you don’t know. Like, has someone…has the whole world seen me naked now?

#### Violence in the Plural

This subtheme captures the notion that exposure to violence is seldom a one-time event, nor is exposure to violence limited to only one type of violence. This third continuum became evident in the girls’ narratives and their background information, i.e., the continuum of accumulated violence. This continuum may be better thought of as a spectrum of experience and refers to the differing quantity of adverse experiences that had clustered around these young girls. Adverse experiences or violence can come in one-point exposures but are more often part of a more extensive history of intertwined experiences of vulnerability. In other words, the girl's experiences of online sexual violence were often accompanied by experiences of offline sexual violence, bullying or harassment, and physical violence and neglect. All the girls were considered poly-victimized, which means that they had more than seven types of violence exposures in their lifetime. For some of the girls, their history of adversities had started at a very young age with severe familial disruptions, massive neglect, or severe bullying in school and lack of proper support from home. For others the adversities had started late and were less pronounced, but still often included neglect and lack of support in combination with some kind of experience of violence such as bullying or sexual assault.

### Making Sense of Online Sexual Violence

This theme refers to how the girls made sense of how it occurred that they had been exposed to online sexual violence. The subthemes were as follows: Fulfilling unmet needs, Feeling pressured and coerced, and Acknowledging curiosity.

#### Fulfilling Unmet Needs

For several of the girls, the sense of lack, or unmet basic needs, that they describe in their backgrounds were framed as a driving force in looking for approval in the online space.

All of the girls had experiences of being ostracized by friends or peers in school at some point in their childhood, and several of them also had difficult upbringings or difficulties in family relationships. The family issues were often about not being properly supported by parents or guardians, such as a neglect of basic needs. Erin, 19 years, described her background as emotionally barren: “I could, or I maybe didn’t get that acknowledgment and support as a small child. I guess I didn’t really get to hear that I was good at anything.”

Siri describes being rejected when calling out for support and acknowledgment of her problems:

Siri (17): I don’t remember how old I was, but I had a hell of a hard time. And I said to my mother like this, mum I’m feeling really really bad and I need help. And she just said, no. You’re just saying that because you want the attention.

A feeling of being excluded, by friends or even society at large, and a sense of low self-worth seemed to be at the core of the drive to look for different kinds of approval in the online space. The girls describe these negative emotions about themselves as contributing to the act of sending nude pictures on request because they wanted to know that they were good enough, that someone out there thought they were pretty. Erin, 19 years, described how she searched online for someone to comment positively on her pictures, and the types of responses she was looking for:

Erin (19): Well, comments about my body sort of. Like, you’re gorgeous. I like your body. I like…you know like that… It's like small things that you just, yeah? Well, maybe that's true then. (…) I needed to hear it.

For some of the girls, the interactions led to more and more exploitation and them doing things that they felt bad about afterward. In these cases, the interactions were described by the girls as being a part of a self-harming behavior that was difficult to stop, and that was rooted in a low sense of self-worth and difficulties with self-assertion. Leila, 19 years, describes her school situation as very difficult, paved with exclusion and bullying, and no support from the adult world:

Leila (19): But then the bullying started there as well, and besides the bullying, I also was excluded. But there they were mean in another way. They were mean with nasty comments, about how I looked. One day I think one of them who was in 9:th grade, the highest grade there, threw iceballs at me, threw, and like poured buckets of water over me and stuff like that, in the hallway. And the teacher just passed by and nobody cared*.*

These experiences came to shape her sense of self-worth, and in her view this was the start of a vicious self-harming circle where the attention she received online first felt helpful, but later became like an addiction with negative consequences.

#### Feeling Pressured and Coerced

There was a significant amount of pressure and nagging involved in sexual interactions online, which the girls had to negotiate. Anna, 17 years, described how sending a naked picture could sometimes be an easier way of getting out of a pressured situation: “Yeah, often you just get sick of the nagging. They nag and nag constantly and then I’m like, ok here you get one then. Like that.”

The nagging could also escalate into more or less explicit threats, which were directed at the integrity and respectability of the girls, in the sense that they involved online publishing or other ways of distributing naked images, meant to cause public shame. Sofie, 18 years: described being warned that the sent picture now was out of her own control: “It was like, yeah you maybe shouldn’t send stuff like that if you’re afraid it will come out. ‘Cause I can send these to whomever I want.’ Sort of.”

Some of the girls had experienced extortion based on previously sent images. This could lead to relationships in which the sender felt more and more entwined with the recipient in a destructive interaction. Erin, 19 years, described feeling stuck in a situation where she did not have any control anymore: “And then you’re just, sending more and more and more and more. You obey them, sort of. It's like, you become like a slave.”

The relationship between the girls and the person requesting pictures or sending dickpics was seen as being a complicating factor in handling unwanted sexual requests or images. Anna described how relationships could take an unexpected turn:

Anna (17): I mean you had become sort of good friends, but after a while, it's like: can I get your picture (nude). And you’re just like, but we’re friends. (…) and you don’t want to break contact because you’re friends and all.

When the sexual contacts were made by friends or acquaintances, they were particularly hard to handle. There was always a threat of withdrawal of the friendship, or turning others against them, if they would confront them or refuse to participate in sexual interactions.

#### Acknowledging Curiosity

Several of the participants also acknowledged a certain curiosity in their online sexual interactions, at least at first. They wanted to know what would happen if they sent pictures, and they had a wish to explore sexual relationships and their sexuality. For Sofie, it was the first time she encountered a boy for a sexual purpose:

Sofie (18): Or it was that way for me. I mean, I felt, I had never talked to a guy, and you really wanted to do that, sort of. And then some friend who had a boyfriend, and like, just ah shit, we did this and that. And you’re damn curious as well, so you want to know what would happen, and you don’t know shit you know. The closest you have come to sex is like watching porn sometimes.

The online interaction could also be framed as a part of a mutual sexual relationship, in which the girls viewed the act of sending nudes as a part of the couple's sex life. Agnes, 17 years, described a mutually trusting relationship where the practice of sending nudes was framed as natural: “Yeah. No, I’ve never sent to anyone…any stranger. The only one I sent to, is my boyfriend. But I mean I know him. So…”

The interactions online could start as something that felt thrilling and new, a sense of exploring. The online space was viewed as new and exciting, and the risks were not yet experienced. Leila, 19 years, described a feeling of freshness and a sense of discovery. On the question if she found the first encounter with social media positive, she simply answered: “I didn’t see anything negative about it at the time.”

### Being Affected by Online Sexual Violence

This theme refers to how the girls expressed the psychological consequences of online sexual violence. Some of the girls disclosed very severe consequences in the form of traumatic symptoms. For others, the effects were more subtle, affecting their assumptions about the world and their view of heterosexuality. This main theme consists of three subthemes, namely *Psychiatric problems*, *A commonplace experience*, and *Coloring the view of men.*

#### Psychiatric Problems

The connections and interactions between the girl's psychiatric problems and the unwanted sexual encounters that they experienced online were varied. Several of the girls acknowledged a connection between parts of their psychological problems that made them need psychiatric services—such as severe self-harming behavior and suicidal thoughts, body image problems such as eating disorders, and mood-related problems—and the exposure to online sexual violence. For some of the girls, the online events were a significant factor behind the psychiatric symptoms that they had sought psychiatric services for. Erin, 19 years, likened the sex she had with men met online with the physical self-harming she had done before:

Erin (19): It's like when you cut yourself, I do it to feel good in the moment. Afterward, you feel like shit.

Interviewer: It's a temporary solution?

Erin: Yeah. And that's what I used the sex for.

The girls also viewed their psychiatric problems as one important reason for ending up in sexual situations online that they did not consent to. In their view, their mental distress contributed in different ways to vulnerable mental states and precarious circumstances, which made them targets for abuse. One of the girls describes how men targeted her because they knew of her vulnerability:

Leila (19): No it didn’t feel ok at the time. I mean he knew that I had problems, and many pictures I took were like, many saw that I had cut myself, and many people knew that I was feeling bad. And they used, abused that as something positive. So they could force me because they knew that I was weak at the time. And often, they saw all the new cuts, on my arms for example. So I just recently cut myself. And some saw it. So I think many people used that. Weak point. They find it, it's like that. All people that had said that I should send stuff like that, they find my weak points. They knew that I wasn’t stable.

The events online also came to further shape the girl's self-image and added a layer of shame and guilt to the already difficult emotional states that the girls were going through. For some of the girls, the online sexual events were described to feed the psychiatric problems that they were already struggling with. Anna, 17 years, had a background of eating disorders and issues with body image. She describes how sexual attention online could strengthen the anorexic part of her, making her feel good about her weight loss:

Anna (17): And then someone said, oh my god you look pretty, and wow what a waist you have. I mean something like that. And you’re like, oh well then I must be thin after all then.

Interviewer: Right, so it's like acknowledging you’re on the right track?

Anna: Yeah, it's like the anorexia gets happy.

The girls expressed the need for staff in psychiatric care to take the issue of online sexual violence into account, as it for some girls was a big part of their psychological distress. It was framed as a world that adults know little about but should become more involved in. Since it could be a way of self-harming, several of the girls highlighted how Child and Adolescent Psychiatric (CAP) clinics should take the phenomenon into account. Erin, 19, years expressed the importance of listening and asking about online experiences and self-harming through sex. On the question of how CAP professionals should approach the subject, Erin answered: “Take it seriously. Because it's nothing to be ashamed of, I mean it's nothing you can just dismiss. It's like any other form of self-harm. And one day everything can go bad. You never know.”

Several of the girls did not acknowledge a causal connection between the reasons they needed psychiatric care and the sexual harassment or abuse they had been exposed to but stated that this area of their life was very important and that the adult world should look into and try to understand their online life better. This was expressed as an important part of why they participated in the study, that this area becomes highlighted.

#### A Commonplace Experience

For many of the girls, the unwanted sexual encounters online seemed to be viewed as so commonplace, that they did not evoke a big response. They did not experience traumatic symptoms in the aftermath, but rather a change in worldview. The girls expressed a kind of cynicism in their view of the online space and the men who inhabited it. The almost constant stream of dickpics (several unique dickpics a week for most of the girls) or other forms of sexual contact via their smartphone, was not framed as a big problem. The girls had become used to it, and they did not express feeling especially bad about it.

Several of the girls framed their experiences as a part of a collective experience, especially for girls their age, which they had to navigate if they wanted to use the social media and devices that were a part of their day-to-day social life. Amelie, 18 years: “That… I mean they, it's not just me, it's like…I don’t know anyone who hasn’t got anything like that.”

The girls seemed to feel that the adult world neglected this part of the online experience, due to its commonality and ubiquitousness. The accounts reflected a kind of boys will be boys’ mentality among adults, that a normal part of being online, as a girl, is to navigate unwanted sexual requests and exposures. On the question of whether online sexual violence is a subject that can be talked about in school, Klara 15 answered: “Sometimes you talk about it. But it's…no, I don’t know. Never. I guess it's so common.”

#### Coloring the View of Men

In the girls’ narratives, it became evident that the constant exposure to unwanted sexual encounters was viewed as something affecting their view of men/boys. Acquiring sex was framed as a top priority for men/boys, and was described as a typically male endeavor, and the girl's unwanted sexual encounters online were uniformly male. On the question of how the different difficulties in her life were connected, Siri, 17 years, answered: “Yeah, I mean … like- it's guys. (…) It's always guys.”

The quantity of unwanted sexual encounters online shaped the girls’ view of men's sexuality, which was seen as more aggressive and hence different from their own sexuality. Mari, 16 years, described how she was affected: “Well, it kind of makes me perceive that boys are only after that kind of thing.”

Sofie, 18 years old, described how her experiences online leaked into her view of the men and boys around her, and that she came to the conclusion that most of the men in her circle of acquaintances had been involved in sexual encounters online that had been unwanted from the female side:

Sofie (18): About most guys I think like: you have probably done the exact same thing, you know.(…) And I think few guys haven’t had a period in their life where they have done that (sending dickpics or requesting nudes). I mean, I think that most girls have sent something sometime. This makes me think that few guys haven’t asked, for people (girls) to send.

On the other hand, some of the girls expressed an attempt to keep men in general out of being associated with the ones sending unwanted sexual material. Anna stressed the possibility of both sexes being exposed to unwanted sexual encounters.

Anna (17): I mean I always support guys, so I never say like, that I feel a lot of people do, that guys never are subjected to any sexual violation or anything like that. I’m always strict about that, that it happens to both [sexes].

Another way of viewing the phenomenon was to add a dimension of pity for some of the men on the other side of the screen. One of the girls described how she could feel sorry for some of the boys/men she encounters online:

Agnes (17): Yeah, sometimes it may be that they…the ones sending pictures tell some sad story. That like, no, but if you, you can’t block me. I don’t have any friends. If you block me I will kill myself. And stuff like that.

Portraying the sender or requester as crazy or deviant, an anomaly that should not be taken seriously could also be a way of framing the events and making them seem less like a part of a structural problem. Klara, 15 years states: “I mean I don’t get sad or take offense if someone sends a dickpic. I mean then, I just block them/erase them. I guess I just think that they are crazy.”

### Resisting Online Sexual Violence

#### Shutting the Door

Some of the girls never interacted with the men that approached them online, while others experienced intense contact, offline meetings, and contact sexual abuse. For all of the girls, the severe abuse situations had stopped, but there was still a constant stream of requests, nagging and coercive behaviors to handle when using online communication tools. To cope with the repeated attempts to engage the girls in interactions, all the girls had eventually found strategies to protect themselves.

All the girls knew about the function in most apps and online social media to block a certain sender from contacting them. This strategy was commonly used by the girls to avoid further contact with strangers sending them disturbing sexual images or requests. Anna, 17 years, described how she got a strong negative emotional response to the dickpics she received, but that this response should never be disclosed to the sender. Instead she just blocks them out: “And then some old man sends a picture of something gross and then you block…I mean you’re like: ‘what the hell are you doing’, and then you like block them.”

The same is described by Amelie, 17 years old: “It was just more uncomfortable to write something back. I don’t want any contact with people like that.”

Siri, 17 years, expressed an unsentimental view of how one should deal with unwanted sexual invitations and dickpics*:* “So if someone starts writing something to me, I just block them. I have never exactly taken offence by it. I just block them and move on with my life.”

The girls seemed to think of this coping-strategy as obvious and did not problematize the fact that it also in a way shifted the responsibility to the girls to shield themselves from different forms of harassment. The blocking seemed to aid the girls in normalizing the occurrence of online sexual violence, contributing to the view of it being a normative experience for a young girl in the age of the internet, and that the soundest way of dealing with it would be to block it out in different ways.

#### Fighting Back

Fighting back against the overwhelming number of dickpics, nagging and distribution of earlier sent images was not easy. One girl described how one way of fighting back was to send back pictures of male friends, as a means of mocking the original sender.

Sofie (18): It's more fun to mock them, for me. And they…it often becomes a pretty difficult situation for them when like, a guy sends back a picture. Then it becomes like, oh f*ck. And then like, yeah, sorry bro, I didn’t know it was a guy*.*

This mocking could be interpreted as a way of playing with gender, turning the hierarchy upside down. It also highlights a heterosexual norm in this gender hierarchy.

Being active and outspoken and sometimes using legal means was another strategy of fighting back. However, the sheer amount of unwanted sexual encounters online could feel overpowering:

Sofie (18): And just like, ‘cause what can you do. You can’t bear it. For a while, I had a period when I wrote back to everyone, like this is not ok and you are violating this and that rule, and this counts as involuntary exposure and bla bla bla. And had like a whole damn rule book in my notes (on the smartphone). But after I while you can’t keep it up. I mean I think most people have a period when you’re like, no this is not ok. You can’t do this. It goes under this paragraph.

A more subtle way of fighting back against online sexual violence was to question the “natural” response of shame when confronted with threats of distributing naked or seminaked pictures. That a girl should need to be ashamed of her body, and why is it shameful to find one's picture on a porn site was questioned by several of the girls. This can be seen as a kind of resistance, questioning gendered norms of sexuality and femininity. One other way the girls fought back was to try informing the adult world about the state of society online. Several of the girls expressed a wish to use their experiences to prevent and help others subjected to online sexual violence. Participation in this research was framed as a kind of social action, to highlight the phenomenon and make society aware. As 18-year-old Amelie puts it after our conversation ended: “I’m glad to be a voice that gets heard.”

## Discussion

The findings of this study are summarized in four main themes. The first main theme suggest that online sexual violence can be conceptualized as happening on several continuums. One that describes the severity of the events, one that captures the intertwinement of the online/offline arenas, and the last that shows the interconnectedness of all violence exposures on a sliding scale from less to more. The advantage of the continuum concept is that it illustrates how things that would otherwise be seen as different phenomena are bound together with a common denominator.

To account for the varying experiences of online sexual violence that the girls in this study experienced, the “continuum of sexual violence”, proposed by Kelly, can be applied. (Kelly, 1987). This concept proposes that all these events share the common denominator of gender inequality and power hierarchies and should therefore all be viewed as serious. From this perspective, one could argue that these events are symptoms of a deeper societal problem rather than only individually experienced.

The sliding scale between online/offline, which is described in the subtheme *Online/offline*—*same but different* illustrates how subjection to violence is not only happening online or offline, but in both modalities (Tamarit-Sumalla et al., 2022). They are often ongoing events that can start in either modality, and continue into the other (Mitchell et al., 2016). Sexual violence shares the same pattern (Svedin et al., 2021). Most of the girls in this study had been subjected to sexual violence both online and offline, and the events were often not clearly circumscribed, but had an ongoing quality. The effects of online sexual violence can thus be hard to define and separate from the effects of IRL-sexual violence. However, the girls did attribute some core features that made the online experience different. A salient part of life online was the possibility to be anonymous, which the girls thought facilitated the abundance of unwanted sexual activities (Yar, 2005). Anonymity as a predictive factor in different forms of online aggression, such as online sexual violence, is a known and discussed phenomenon referred to as the online disinhibition effect (Suler, 2004). Another attribute of the online modality was its vastness, and a loss of control of what is published. A fear of photos and films resurfacing, and the lack of control of the material as a distinctive feature of the online modality, has been expressed by both girls in this study and participants in other qualitative studies (Hamilton-Giachritsis et al., 2020).

The third facet of the continua, *Violence in the plural*, illustrates the fact that violence often clusters around individuals. All the girls were as poly-victimized, and online sexual violence was one part of a larger web of violence (Hamby, 2013). This pattern is shown in a large body of research and is important to take into account when researching psychiatric symptoms in girls subjected to online sexual violence (Finkelhor et al., 2007).

The following three main themes describe and illustrate the girls’ own perceptions about the events, from several different angles. In the theme *Making sense of online sexual violence*, we describe how the girls explained their exposure to online sexual violence. They attributed it to several factors. In the first subtheme, *Fulfilling unmet needs*, the girls describe a feeling of unfulfilled basic needs, such as nurture or acknowledgement. This lack was in their view causing low self-esteem and a need to seek approval online. A sense of low self-worth or low self-esteem as the driving force for girls to be sexually active is a discourse that has been reproduced by girls in other studies, see for example (Wilkins & Miller, 2017). In this discourse, girls’ sexual agency is neglected, and the concept of desire is missing (Fine, 1988). When the girls attribute their longing for approval to an individual lack of fulfilled personal needs, the link to culture is minimized or left out. The feeling of lack, that is sought to be fulfilled by boys’ affirmation of girls’ attractiveness, or by boys attending to them, is of course partly caused by a real lack of nurture and acknowledgement in their living environment. However, it is also due to a cultural pressure. Social norms of desirability urge girls to produce sexiness for boys to get their approval and this social pressure is important to consider when theorizing about causes of online sexual violence (Ringrose et al., 2013).

Actual pressure or coercion from boys to engage in sexual activities was, not surprisingly, also brought forth as an explanation for why the girls had experienced online sexual violence. This is expressed in the second subtheme, *Feeling pressured and coerced*. All the girls had experiences of being pressured to send naked pictures of themselves using apps on their smartphones. Other studies of this phenomenon show that pressuring girls to send naked photos of themselves and collect these as trophies can be a part of a homosocial and heteronormative bonding practice for boys (Ringrose et al., 2022). A recent Swedish report from ECPAT highlights discourses of gendered responsibility in online sexual violence (Andersson, 2022). The boys in the report rejected responsibility for how they interacted with girls to acquire naked photos and put the responsibility on the girls. A tendency for boys to ignore male perpetration and hold girls accountable is also seen in other studies (Salter, 2016). The fact that girls need to consider the risk of being shamed by the community for sending sexual photos has been expressed in other qualitative studies (Ringrose et al., 2022). So-called “slut shaming” has long been a part of the sexual regulation of femininity and girls’ and women's bodies (Ringrose & Harvey, 2015). In line with the sexual double standard, girls are called upon to engage sexually with boys but are also punished for engaging “too much” (Ringrose et al., 2013). The responsibility for naked material ending up in the wrong hands is also placed with the girls, rather than with the ones distributing it (Salter, 2016).

Although many of the explanations for online sexual violence touch upon victimhood and perpetration, this is not the whole story. A part of the girl's involvement in being online and sending nudes was also due to their curiosity and excitement in exploring their sexuality. This is illustrated in the subtheme *Acknowledging curiosity*. In general, sexual agency and sexual desire are lacking in the societal discourse about adolescent girls. They are often viewed as victims or at risk of being victims and are left without narratives of female sexual agency and desire (Egan & Hawkes, 2008; Meehan, 2021). This is even more so when it comes to girls with mental health issues, where their involvement in sometimes sexually risky situations can often be framed as a consequence of their psychiatric difficulties. This can lead to the lack of guidance from the adult world in a positive exploration of girls’ sexuality. It is important to lift this issue, to not reproduce girls as only victims, but also in part agents, in the narratives around sexuality and girls.

The third theme, *Being affected by online sexual violence,* is conceptualizing the different ways that the girls had been affected by exposure to online sexual violence.

The first consequence was psychiatric symptoms, or that their psychiatric problems interacted with the exposure to online sexual violence in negative spirals. This is illustrated in the subtheme *Psychiatric symptoms*. The connection between mental health, often conceptualized as trauma, and online sexual violence has been clearly shown in several studies (Jonsson et al., 2015, 2019; Svedin & Jonsson, 2017). Shame and a negative view of the self is often at the core of these problems (Joleby et al., 2020). It is also shown that girls with psychiatric disorders are exposed to more violence in all forms, but especially sexual violence (Svedin et al., 2023). It is thus not surprising that several of the girls expressed a synergic relationship between their psychiatric problems and their exposure to online sexual violence. Psychiatric problems can be seen as both a cause and an effect of online sexual violence, interacting in negative spiraling ways. The role of shame in these girls’ psychiatric problems was clear, and this is something described in other literature as well (Henry & Powell, 2015; Joleby et al., 2020). Deconstructing this shame as a gendered discourse is important when addressing online sexual violence among psychiatric patients (Weiss, 2010). Learning to see sexual violence as a symptom of gender inequality has been shown as a way of buffering this shame (Strauss Swanson & Szymanski, 2022).

However, being subjected to online sexual violence was not only seen as traumatic or causing psychiatric symptoms, but it was also viewed as a normative experience for girls in general in this day and age. This is illustrated in the subtheme, *A commonplace experience*. Here the girls describe the normalization process of violence sparked by the sheer quantity of the experiences. The girls expressed that the unwanted sexual encounters online were a part of an everyday experience, and several of them did not frame themselves as victims of sexual violence per se. That women see their experiences of sexual violence as negative, but do not want to position themselves as victims have been shown in other studies (Amundsen, 2021). The experience of online sexual violence can thus be viewed as a normative experience for girls and women, constituting their life circumstances as female gendered. At the same time, it also causes, and interacts with, severe psychiatric difficulties such as self-harming or depressive symptoms. Normalization of violence is a process, known form other research of violence, that serves to numb the victim and allow for the continuation of violence perpetration (Aghtaie et al., 2018).

Another important way the girls were affected by online sexual violence was that it colored their view of men, relationships, and heterosexuality in a way that emphasized and normalized stereotypical masculinity. In the subtheme *Coloring the view of men*, the girl's accounts reproduced discourses about male aggressive sexuality as normal and expected. This is a pattern that is shown in other studies of sexual violence victims (Weiss, 2009). This subtheme ties closely in with the normalization of sexual violence altogether, and to the mostly passive ways of coping with these events that are illustrated in the last main theme, *Resisting online sexual violence*.

When resisting online sexual violence, the girls had two main ways of coping with the ubiquitous unwanted sexual interactions online. By *Shutting the door,* which mainly consisted of the practice of blocking out senders via the digital application's functions, the girls could shield themselves from unwanted sexual attention. This practice was the most common and framed as obvious and non-problematic by the girls. However, the ability to block out the perpetrators also seemed to aid in the normalization of the events, and to keep the girls from viewing the acts as victimizations and organizing themselves or fighting back.

Some of the girls did resist in a more active manner, which is illustrated with the subtheme *Fighting back.* By reporting what happened, informing the sender of rules and legislations, or fighting fire with fire by paraphrasing the senders’ pictures in some humorous way, the girls could stand their ground and feel more empowered. Other research has delved into humor as way of resisting online sexual harassment, thus counteracting the narrative about internet as only risky for women and girls (Vitis & Gilmour, 2017). The girls in our study also described a more subtle resisting, by questioning the sexual double standard of female sexuality and the shaming of naked bodies that serves to suppress girls’ sexuality. These resisting discourses have been shown in other research with adolescent girls (Jackson & Cram, 2003).

## Conclusion

As the findings suggest, online sexual violence constitutes a multifaceted experience along several continuums for girls in need of psychiatric services. It is interwoven with psychiatric distress and other violence exposures and is best thought of as an ongoing phenomenon rather than circumscribed events.

From a gender perspective, the findings also show how girls in psychiatric care need to navigate societal norms about gender and sexuality in the same ways as girls in general. Online sexual violence can thus be thought of as a common girl experience, as well as having consequences that need tending to in the framework of psychotherapy or psychiatric intervention. This embeddedness in social circumstances of the problems and issues of girls in psychiatry should be considered in treatments and psychotherapy. From a psychiatric point of view, it is important to be aware of the norms and discourses that affect the views that psychiatric staff hold about what it means to be a girl, what causes mental distress in girls, and female adolescent sexuality. Gender norms can cause blind spots among psychiatric staff, affecting which areas of girls’ lives should and should not be addressed in therapy. These norms can color assessments and treatments for girls with experience of sexual violence.

## Limitations

The study describes the experiences of young girls in psychiatric care with backgrounds that include of some kind of online sexual violence. The experiences where varied, and the experiences of specific events of online sexual violence were therefore not explored in depth. The phenomenon was investigated from the perspective of girls in the age of 15–20 with psychiatric care needs, which could be a limitation to the transferability of the findings.

Two of the authors are working clinically in child- and adolescent psychiatry. This is both a strength and a limitation. Strength because it enables prolonged engagement with the group and a thorough knowledge of the group's problems and experiences, and a limitation because it might also cause blind spots in interpretations. To solve this problem, we have used reflexivity throughout the process.

## Suggestions for Future Research

Further research in this group of young people should explore specific experiences within the broader umbrella of online sexual violence, such as specifically grooming or pressurized sexting.

To further enhance knowledge about the phenomenon of online sexual violence in psychiatry, efforts should also be made to broaden the inclusion criteria and interview also boys and sexual minorities in psychiatric care with relevant experiences. Another important topic to look further into is the experience of online sexual violence among younger children with psychiatric care needs.

Future research should moreover look further into how girls with psychiatric care need can be supported in developing and exploring their sexuality, while at the same time keeping safe from sexual violence, online as well as offline. Another topic that should be further explored and deepened is how shame plays a role in the development and maintenance of psychiatric symptoms after online sexual violence, and how this shame is connected to gender norms of sexuality.
